# The Gingival Oral Lichen Planus: A Periodontal-Oral Medicine Approach

**DOI:** 10.1155/2019/4659134

**Published:** 2019-01-06

**Authors:** Abdulhameed Alsarraf, Kunj Mehta, Nabil Khzam

**Affiliations:** ^1^Ministry of Health, Kuwait; ^2^Private Practice, Perth 6000 Western Australia, Australia; ^3^NK Perio & Implants, Perth 6172 Western Australia, Australia

## Abstract

We present a case of a 77-year-old female who suffered from oral lichen planus (OLP) involving her gingiva and bilateral buccal mucosa for over 6 months. We showed that oral hygiene measures and conventional periodontal treatment and strict maintenance were sufficient to control the gingival involvement of OLP. The mechanism of OLP is complex and not yet fully understood. The focus of discussion in our case was that knowledge and understanding of gingival pathology are fundamental for a determined management approach. Our case was managed according to the suggested protocols in previous case studies. A multidisciplinary approach allowed for accurate diagnosis and treatment tailored to the presented case.

## 1. Introduction

Lichen planus is a chronic immune-mediated inflammatory disease that affects the skin and mucous membranes. Oral lichen planus (OLP) is the mucosal counterpart of cutaneous lichen planus and it was first described in 1866 as white papular eruptions in the oral cavity [1]. According to the American Academy of Periodontology, OLP is classified as nonplaque-induced gingival lesions [2]. Classically, OLP appears in a roughly symmetrical distribution of well-defined white striations on a background of mild erythema, commonly involving the buccal mucosa and tongue [3]. Other clinical patterns reported in the literature include atrophic, erosive, papular, plaque-like, and bullous-type lesions [4]. To ensure accurate diagnosis of OLP, key histopathological features from biopsy specimens should be reported by an oral pathologist to allow clinicopathological correlation and exclusion of oral epithelial dysplasia. The pathogenesis of the disease is characterized by cytotoxic CD8^+^ T lymphocytes migration to the epithelium inducing apoptosis of basal keratinocytes [5]. A number of etiological factors in OLP have been proposed such as local and systemic inducers of cell-mediated hypersensitivity, drugs, dental materials, infectious agents, and stress [6]. OLP management is directed at controlling active inflammation and the reduction of associated symptoms; therefore, patient education and awareness is important [7]. In addition, maintaining good oral hygiene, absence of oral candidosis, periodontal disease, and salivary gland hypofunction are all factors to successful management outcomes [8]. Various therapeutic agents are described for controlling OLP including topical, intralesional, and systemic corticosteroids, immunosuppressive agents, retinoids, and immunomodulators. These treatment regimens may be used sequentially until effective symptomatic control is reached due to variations in patient responses [9–11]. Of note, the malignant potential of OLP is reported in the literature with an overall malignant transformation of 1%. This is due to cases of oral cancer arising from patients with OLP demonstrating absence of epithelial dysplasia [12, 13]. OLP has therefore been designated as an oral potentially malignant disorder. It must also be noted that similar clinical presentations of OLP are induced by oral lichenoid reactions; however, it is not always simple to distinguish between the two different diseases clinically as well as histopathologically [9]. With that being said, most oral lichenoid lesions are directly presented adjacent to direct restorative materials such as amalgam and composite [9]. Further understanding and clinical experience aids in implementing a long-term management plan for OLP cases.

## 2. Case Report

**Figure 1:**
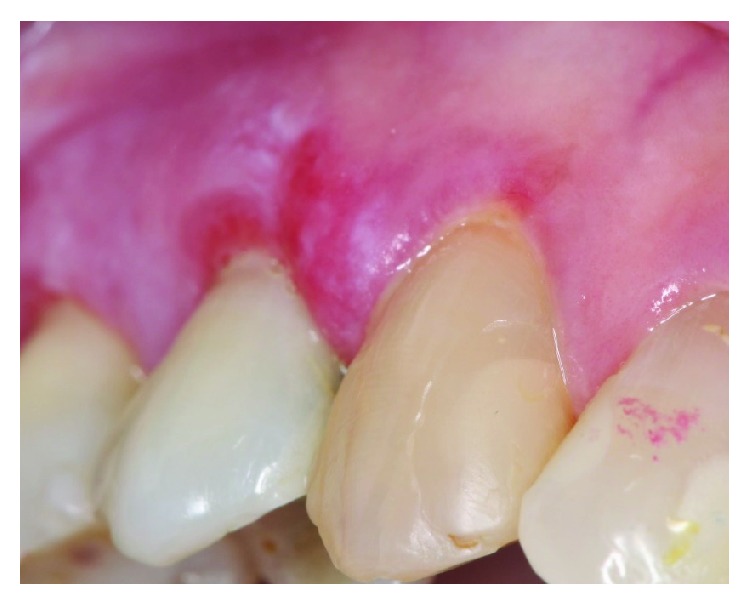
Gingival OLP: a mixed red and white lesion in the background of white striations involving the gingiva between teeth 13 and 14. No to minimal plaque is noted and the involvement of the attached gingiva distinguishes OLP from plaque-induced gingivitis.

**Figure 2:**
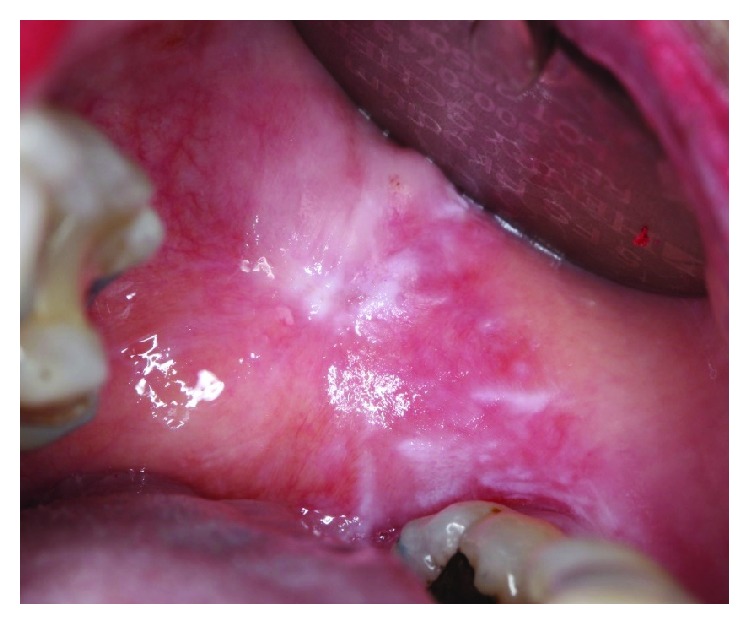
Left buccal mucosa: predominantly white plaque-type lesion and white striations noted in the background of mild erythema.

**Figure 3:**
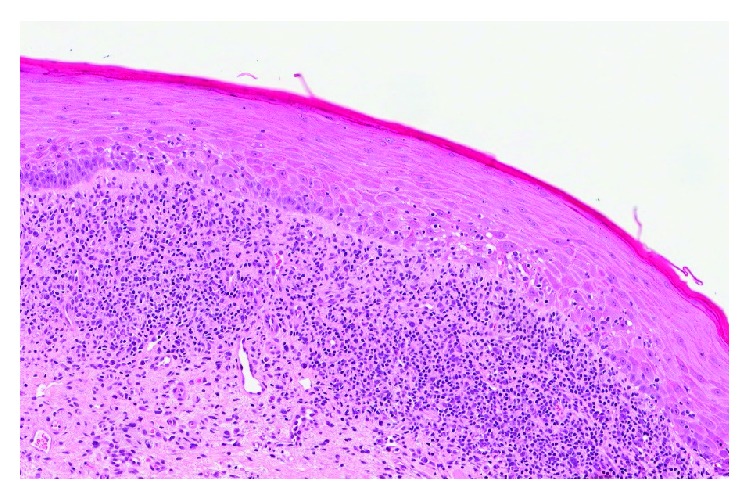
Histopathological assessment from an incisional biopsy performed from the left buccal mucosa showed hyperkeratosis and band-like lymphocytic infiltrate in the lamina propria.

A 77-year-old female patient presented to her general dentist due to bleeding gums. The dentist referred the patient to a specialist periodontist for a consultation regarding the assessment and treatment of generalized chronic periodontitis. A full comprehensive periodontal and radiographic examination revealed a periodontal diagnosis of generalized moderate to advanced chronic periodontitis. Clinical signs of gingival inflammation and periodontal pockets of 5 mm and more with calculus and bleeding upon probing were present on two or more aspects of each tooth. The radiographic examination revealed a generalized horizontal bone loss of 40 to 50% around most of the dentition. The patient was then referred to the Oral Medicine Clinic for diagnosis and further management of OLP-like lesions. Incisional biopsies were performed from the left buccal mucosa and 13/14 labial gingiva (Figures 1 and 2). Histopathological assessment showed hyperkeratosis and band-like lymphocytic infiltrate in the lamina propria (Figure 3). No epithelial dysplasia was noted. These features are consistent with the diagnosis of OLP. Patient education and awareness was delivered in the context of diagnosis, potential triggering factors, and disease malignant potential. Long-term observation is necessary, and the patient will be followed up regularly to monitor disease behavior and progression.

## 3. Discussion

**Figure 4:**
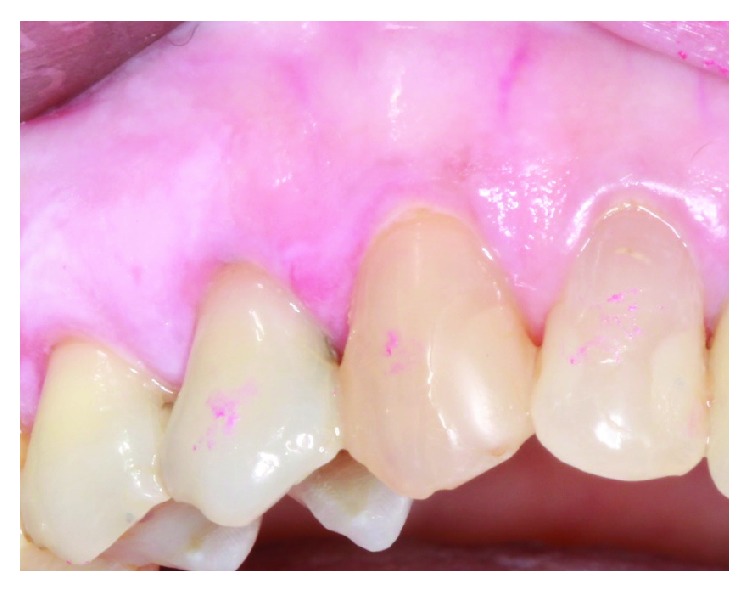
Resolution of erythema and active gingival inflammation due to OLP lesion involving the gingiva between teeth 13 and 14 following use of topical corticosteroid ointment.

The diagnosis of OLP requires both clinical manifestations and characteristic histopathological features. The practice of good oral hygiene and achieving optimum periodontal health contribute to successful management outcomes. From a clinical standpoint, the proposed periodontal treatment plan was patient education, in the form of information on periodontitis in terms of pathogenesis, treatment, and prevention. Detailed oral hygiene instructions include recommendations for the use of electric ultrasoft tooth brush, spongy dental floss, and soft interdental brushes. The patient was encouraged to maintain high level of oral hygiene and attend regular periodontal review appointments in order to maintain periodontal stability. The patient underwent two sessions of nonsurgical periodontal therapy on the same month, followed by reevaluation of periodontal tissue conditions 12 weeks after completion of debridement. This was followed by supportive periodontal treatment (3 monthly intervals). Overall, the patient had an excellent response to the nonsurgical periodontal therapy with good oral hygiene. In view of the involved OLP, additional attention is required in the overall management of the case. OLP is frequently asymptomatic or associated with minor discomfort only. This is especially true for papular, reticular, and plaque-type lesions which are often unnoticed by the patient and the general dentist. In contrast, atrophic and ulcerative lesions may give rise to severe pain, particularly related to food intake and aggressive oral hygiene procedures. In the presented case, the OLP lesion involving the gingiva of teeth 13 and 14 was symptomatic; therefore, the patient was instructed to use a topical corticosteroid ointment three times daily for two weeks applied directly to the lesion. Specifically, betamethasone valerate 0.05% was used as a topical application to the affected areas. Successful resolution of background erythema and active gingival inflammation was noted in the review appointment (Figure 4). Other areas involving the oral cavity such as the left buccal mucosa improved significantly. From a periodontal perspective, the most important part of the therapeutic regimen is treatment of periodontal disease (conservative nonsurgical approach), an atraumatic oral hygiene in terms of gentle tooth brushing and flossing using spongy type floss. Evidence of the literature supports this statement in the context of personalized plaque control for OLP gingival manifestations [14]. This will in turn result in significant improvement in a large number of patients. Effective plaque removal without traumatic influence on the gingival tissues must be established. In case of persistent pain typically associated with ulcerative and atrophic types, antifungal treatment may be necessary if the lesion contains candida species [8]. In chronic and symptomatic OLP cases, the response to the above treatment regimens is usually poor; therefore, topical corticosteroids are considered the first-line medications to control patients' symptoms. However, relapse in such cases is very common and intermittent episodes of treatment may be required over an extended period of time.

## 4. Conclusion

This case has demonstrated the periodontal management of a patient with oral lichen planus. While oral hygiene measures and conventional periodontal treatment were sufficient to control the gingival involvement of OLP, patient education and awareness of triggering factors were important to control OLP activity with the aid of topical corticosteroids. It is however important to consider the side effects related to long-term use of topical corticosteroids in the oral cavity. It is therefore important that both knowledge and understanding of gingival pathology are fundamental for a determined management approach in such cases.
